# A Novel Fixed Low-Rank Constrained EEG Spatial Filter Estimation with Application to Movie-Induced Emotion Recognition

**DOI:** 10.1155/2016/6734720

**Published:** 2016-08-11

**Authors:** Ken Yano, Takayuki Suyama

**Affiliations:** Department of Dynamic Brain Imaging, Cognitive Mechanisms Laboratories, Advanced Telecommunications Research Institute International, 2-2-2 Hikaridai, Seika-cho, Soraku-gun, Kyoto 619-0288, Japan

## Abstract

This paper proposes a novel fixed low-rank spatial filter estimation for brain computer interface (BCI) systems with an application that recognizes emotions elicited by movies. The proposed approach unifies such tasks as feature extraction, feature selection, and classification, which are often independently tackled in a “bottom-up” manner, under a regularized loss minimization problem. The loss function is explicitly derived from the conventional BCI approach and solves its minimization by optimization with a nonconvex fixed low-rank constraint. For evaluation, an experiment was conducted to induce emotions by movies for dozens of young adult subjects and estimated the emotional states using the proposed method. The advantage of the proposed method is that it combines feature selection, feature extraction, and classification into a monolithic optimization problem with a fixed low-rank regularization, which implicitly estimates optimal spatial filters. The proposed method shows competitive performance against the best CSP-based alternatives.

## 1. Introduction

Brain computer interfaces (BCIs) are a rapidly growing field of research that combines neurophysiological insights, statistical signal analysis, and machine learning. BCIs are generally designed based on a pattern recognition approach, that is, extracting features from EEG signals and using a classifier to identify the user's mental state from such features [1]. Those sequential approaches are called “bottom-up” schemes; given a large collection of single-trial EEG data, better representations of the data are extracted to finally obtain the classification output at the top. In contrast, discriminative or “top-down” approaches focus on predicting user intentions and are based on two criteria: the empirical prediction performance and the regularizer. Suitably chosen regularizers automatically induce sparse decomposition of the signal, which corresponds to conventional feature extraction [2].

This paper proposes a discriminative method using a low-rank regularizer to estimate spatial filters for extracting effective features under a study. The advantage of the proposed method is that it combines feature selection, feature extraction, and classification into a monolithic optimization problem with a low-rank regularization, because this approach includes spatial filter estimation in the optimization framework of statistical inference model. Under a suitable chosen regularizer, it induces the best inference model, which implicitly estimates optimal spatial filters under the regularization assumption.

Emotion classification from EEG data has attracted much attention recently [3, 4]. Emotion also plays an important role in human-human communication and interaction. The ability to recognize the emotional states of people is an important part of natural communication. This field of research is still relatively new, and there is still much to be done to improve on existing elements in BCI but also to discover new possibilities.

For evaluation of the proposed methods, experiments were conducted to induce emotions by movies for dozens of young adult subjects and estimated the emotional states using the proposed method. The results were compared with conventional methods using a common spatial pattern (CSP).

This paper's contribution is the proposal and the explicit derivation of the fixed low-rank constrained discriminative approach and its application to emotion recognition with comparative analysis with conventional methods. This paper is organized as follows.  Section 2 describes the background of emotion recognition from EEGs, and Section 3 describes the proposed method.  Section 4 presents the data acquisition and experimental protocol.  Section 5 describes the results and discussion.  Section 6 concludes the paper.

## 2. Background

### 2.1. Emotion in the Brain

The limbic system which is like a cortical ring around the brain stem is responsible for initial emotional interpretation of the signals from the autonomic nervous system. The hypothalamus is responsible for processing the incoming signals and triggering the corresponding visceral physiological effects like a raised heart rate or galvanic skin response.

From the hypothalamus the stimuli information is passed on to the amygdala, which is important for learning to connect stimuli to emotional reactions (reward/fear) and for evaluating new stimuli by comparing them to past experience.

The amygdala is considered vital for emotion processing. However, since it is an underlying structure like the rest of the limbic system, it cannot be detected directly in recording from the scalp. The amygdala is connected to the temporal and prefrontal cortices, which is thought to be the way visceral sensations are interpreted cognitively, resulting in a consciously experienced feeling of an emotion [5].

The temporal lobe is essential for hearing, language, and emotion and also plays an important role in memory. The prefrontal lobe is involved in the so-called highest level of functioning. It is responsible for cognitive, emotional, and motivational processes. The prefrontal lobe is part of the frontal cortex, which is said to be the emotional control center and to even determine personality. It is involved in, among others, judgment and social behavior. These functions are very much based on the experience of emotions.

### 2.2. Valence: Hemispherical Asymmetry

Psychophysiological research has shown the importance of the difference in activation between the two cortical hemispheres in the reaction that subjects show toward stimuli. Left frontal inactivation is an indicator of a withdrawal response, which is often linked to a negative emotion. On the other hand, right frontal inactivation is a sign of an approach response, or positive emotion.

Harmon-Jones [6] suggests that the hemispherical differences are not an indication of affective valence, but of motivational direction (approach or withdrawal behavior to the stimulus). Affective valence does seem tightly linked to motivational direction. Therefore, the hemispherical asymmetry patterns do indicate the affective valence.

Davidson and Fox [7] found that 10-month-old infants exhibited increased left frontal activation in response to a film clip of an actress generating a happy facial expression as compared to a sad facial expression. Frontal cortical activity has been found to relate to facial expressions of positive and negative emotions as well.

## 3. Method

### 3.1. General Framework

Given a short high-pass filtered EEG segment, *X* ∈ *ℛ* ^*C*×*T*^, where *C* is the number of channels and *T* is the number of time points, the data are first band-pass filtered at a band range being studied. A commonly used form of a second-order or power oscillation-based linear model can be written as follows:

(1) $$\begin{array}{c} f=b+\overset{J}{\underset{j=1}{\sum }}\theta_{j}\log\left(\;\mathrm{Var}\left(\;w_{j}^{T}X\;\right)\;\right). \end{array}$$

Here, {*w*_*j*_}_*j*=1_^*J*^ ∈ *ℛ* ^*C*×*J*^ is the spatial filters, {*θ*_*j*_}_*j*=1_^*J*^ are the weighting coefficients of the *J* features, and *b* is a bias term. The classifier first projects the signal by *J* spatial filters. Next, it takes a logarithm of the power of the projected signal. Finally it linearly combines these *J* dimensional features and adds bias.

To determine spatial filters {*w*_*j*_}_*j*=1_^*J*^, CSP is often used [1]. Many variants of the original CSP have been proposed. [8]. Coefficients {*θ*_*j*_}_*j*=1_^*J*^ and *b* are determined statistically from the training examples, that is, the pairs of trials and labels {*X*_*i*_, *y*_*i*_}_*i*=1_^*n*^ collected in the calibration phase. Label *y* ∈ {+1, −1} corresponds to the binary classes being studied.

To briefly summarize CSP to compute spatial filter *w*, it is obtained by extremizing the following function:

(2) $$\begin{array}{c} J\left(\;w\;\right)=\frac{w^{T}\Sigma_{\left(+1\right)}w}{w^{T}\Sigma_{\left(-1\right)}w}, \end{array}$$

where Σ_(*c*)_ is the spatial covariance matrix of the EEG signals from class *c* as follows:

(3) $$\begin{array}{c} \Sigma_{\left(c\right)}=\frac{1}{\left|I_{c}\right|}\underset{i\in I_{c}}{\sum }X_{i}X_{i}^{T}\;\left(c\in +1,-1\right), \end{array}$$

where we assume a zero mean for the EEG signal.

Since *J*(*w*) remains unchanged if *w* is rescaled, extremizing *J*(*w*) is equivalent to extremizing *w* ^*T*^Σ_(+1)_ *w* subject to the constraint *w* ^*T*^Σ_(−1)_ *w* = 1. Using the Lagrange multiplier method, this constrained optimization problem amounts to extremizing the following function:

(4) $$\begin{array}{c} L\left(\;\lambda ,w\;\right)=w^{T}\Sigma_{\left(+1\right)}w-\lambda \left(\;w^{T}\Sigma_{\left(-1\right)}w-1\;\right). \end{array}$$

The spatial filter *w* extremizing *L* is such that the derivative of *L* with respect to *w* equals 0:

(5) ∂L∂w=2wTΣ+1−2λwTΣ−1=0⟺Σ+1w=λΣ−1w⟺Σ−1−1Σ+1w=λw.

The spatial filters are the eigenvectors of Σ_(−1)_^−1^Σ_(+1)_ which correspond to its largest and lowest eigenvalues.

### 3.2. Proposed Model Calibration

If we ignore the logarithm in (1), it can be reformulated as follows:

(6) $$\begin{array}{c} \overset{J}{\underset{j=1}{\sum }}\theta_{j}\left(\;w_{j}^{T}XX^{T}w_{j}\;\right)=\mathrm{Tr}\left(\;\Theta^{T}\Sigma \;\right), \end{array}$$

where Θ = ∑_*j*=1_^*J*^ *θ*_*j*_ *w*_*j*_ *w*_*j*_^*T*^ ∈ *ℛ* ^*C*×*C*^ and Σ ∈ *ℛ* ^*C*×*C*^ is the covariance matrix of *X*. Finally we obtain

(7) $$\begin{array}{c} f=b+\langle \;\Theta ,\Sigma \;\rangle . \end{array}$$

Note that 〈Θ, Σ〉 is the elementwise inner product of the two matrices. To determine parameters (Θ, *b*), logistic regression was employed with low-rank regularization of Θ. This amounts to solving the following optimization problem with training examples:

(8) minΘ,b ∑i=1nlog⁡1+e−yib+Θ,Σi+∑i=1cσis.t. rank⁡Θ=c,

where *σ*_*i*_ is the *i*th singular value of Θ and *c* is the rank constraint of Θ. The first term is convex. But since the low-rank constraint term is nonconvex, it is not guaranteed to find the optimal point. To solve this problem, the alternating direction method of multipliers (ADMM) [9] is employed with a hope that it has better convergence properties than other local optimization methods. For nonconvex problems, depending on the initial values, the solution can converge to different points.

The optimization problem is rephrased as follows:

(9) minimize FΘ,bsubject  to Θ∈C,

where *C* is the set of matrices with rank *c*. To solve it by ADMM, it can be rewritten as follows:

(10) minimize FΘ,b+GΞsubject  to Θ−Ξ=0,

where *G* is the indicator function of *C*. The augmented Lagrangian (using the scaled dual variable) is

(11) LρΘ,b,Ξ,Υ=FΘ,b+GΞ+ρ2Θ−Ξ+Υ22,

where *ρ* > 0 is called the penalty parameter. So the iterative optimization of ADMM for this problem is

(12) Θk+1=arg minΘ,b⁡FΘ,b+ρ2Θ−Ξk+Υk22,Ξk+1=ΠCΘk+1+Υk,Υk+1=Υk+Θk+1−Ξk+1,

where Π_*C*_ is the projection onto *C*. Hence, Π_*C*_(Ξ) is determined by carrying out a singular value decomposition, Ξ = Σ_*i*_ *σ*_*i*_ *u*_*i*_ *v*_*i*_^*T*^, and keeping the top *c* singular values; that is, Π_*c*_(Ξ) = ∑_*i*=1_^*c*^ *σ*_*i*_ *u*_*i*_ *v*_*i*_^*T*^.

Here we can initialize Θ and *b* as zero w.l.o.g. The primal and the dual residuals at iteration *k* + 1 are defined as follows:

(13) Rk+1=Θk+1−Ξk+1,Sk+1=−ρΞk+1−Ξk.

These residuals converge to zero as ADMM proceeds.

### 3.3. Multiple Frequency Bands

The proposed method can be extended for estimating the spatial filters for multiple frequency bands. Let *X*_*b*,*k*_ = *B*_*k*_ *X* be the band-pass filtered data by filtering operator *B*_*k*_. The covariance matrix of the signal denoted as Σ_*b*,*k*_ = *X*_*b*,*k*_ *X*_*b*,*k*_^*T*^ ∈ *ℛ* ^*C*×*C*^ is obtained separately for each frequency pass band. Then align them as a large block diagonal matrix (14). To obtain the spatial filters for multiple bands, this block diagonal matrix is substituted for Σ in (7). The solution is expected to effectively select the optimal spatial features from multiple frequency bands:

(14) $$\begin{array}{c} \Sigma =\left(\begin{array}{cccc} \Sigma_{b,1} & & & \\ & \Sigma_{b,2} & & 0\\ 0 & & \ddots & \\ & & & \Sigma_{b,K} \end{array}\right). \end{array}$$

### 3.4. Merits of the Proposed Method

CSP estimates spatial filters based on a criterion that corresponding components produce minimum variance for one condition and maximum variance for the other and thus increase discriminative ability. However because the spatial filter estimation is decoupled from the inference model, such as logistic regression, optimal filters can only be predicted by using cross-validation of the inference model and select the one which produces the best empirical inference performance.

On the other hand, our proposed model derived from “top-down” approach incorporates spatial filter estimation in the predictive model. Hence by focusing on the prediction performance with suitably chosen regularizer, such as fixed low-rank in our model, it induces sparse decomposition of the signal which corresponds to conventional feature extraction. Hence, it implicitly estimates optimal spatial filters of the best inference model under the assumption.

## 4. Emotion Recognition

To predict the state of emotion experienced by the participants from single EEG segments, a predictive model was employed that estimates from a given short EEG segment (here 5 sec) the probability that the participant experienced positive or negative emotions during that period. For the evaluation, fivefold cross-validation is performed by holding out one-session dataset for the test and the remaining four-session datasets with labels were used to estimate parameters (Θ, *b*). For each round, the held-out dataset was used for tests to evaluate the classification error rate. In each round, the classification error rate is computed as the ratio of the number of correctly classified EEG segments divided by the total number of EEG segments in the trial.

### 4.1. Data Acquisition

**Figure 1:**
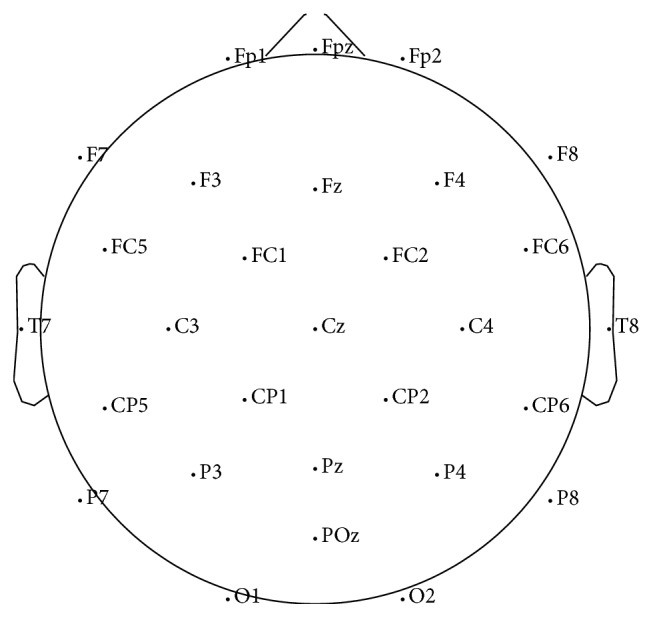
EEG channel locations. For decoding emotions, we use all channels except Fp1, Fpz, and Fp2.

**Figure 2:**
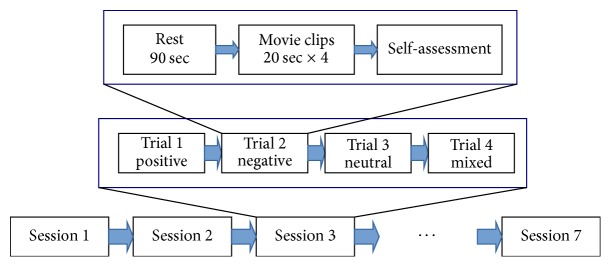
Protocol of the EEG experiment. For each session, we randomly changed the sequence of trials of four movie clip types: positive, negative, neutral, and mixed.

Twenty-three healthy adult volunteers participated under the informed consent that was approved by the ethical committee of ATR. Among them, ten subjects (males = 3, females = 7, age = 24.5 ± 6.24) were selected for analysis. The EEGs were recorded from 32 gel-based scalp electrodes, as shown in Figure 1, and four EOG placements around the eyes using an eego amplifier (ANT Neuro, Enschede, Netherlands) with 24-bit resolution. The EEGs were sampled at 512 Hz. The protocol of the EEG experiment is described in Figure 2. To elicit emotions, a set of movie clips that were used in Samson et al. [10] was used. The movie clip set includes four classes of different target emotional states: positive, negative, neutral, and mixed. The average length of each clip was about 20 seconds. For each trial, to elicit emotions, four randomly selected movie clips of the same emotional class were played continuously without intervals and followed by self-assessment questions. One session consisted of four trials of four different movie classes. Before each trial, a random color grating pattern was displayed for 90 seconds to wash out the emotional states of the participant. The entire experiment consisted of seven sessions. For the analysis, however, only the first five sessions were used because, during the last two sessions, most participants appeared fatigued or drowsy.

### 4.2. Preprocessing

The EEG signals were downsampled from 512 to 128 Hz and high-pass filtered at 0.5 Hz. The EOG and the muscle artifacts were automatically removed using AAR [11]. Among the 32 channels, only 26 channels were used excluding the reference and prefrontal channels, Fp1, Fpz, and Fp2, which were contaminated severely by the EOG artifacts. The EEG signals were rereferenced by the M1 and M2 means. All the trial data were extracted from the onset of the first movie clip until the offset of the last clip. Then all the trial data were band-pass filtered at 4–47 Hz. Finally, the length of all the trial data was identically set to 80 seconds. Training and testing data were generated by using a sliding window over each bit of trial data. The length of the window was five seconds, and the overlap between windows was two seconds.

## 5. Results

**Figure 3:**
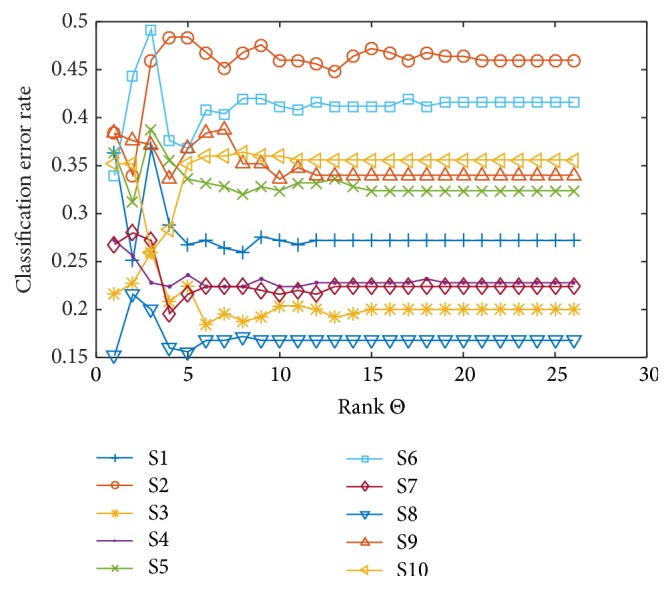
Classification error rate of binary classes (positive or negative) for all subjects. Mean classification error rate changed by increasing low-rank constraint from 1 to 26 (full-rank).

Figure 3 shows the variabilities of the classification error rate for all participants due to the change of the rank constraints. The classification error rate was computed by averaging over folds. It reached plateau after some rank constraints. This figure suggests that an optimal rank constraint exists between 1 and 10 regardless of the participants.

**Figure 4:**
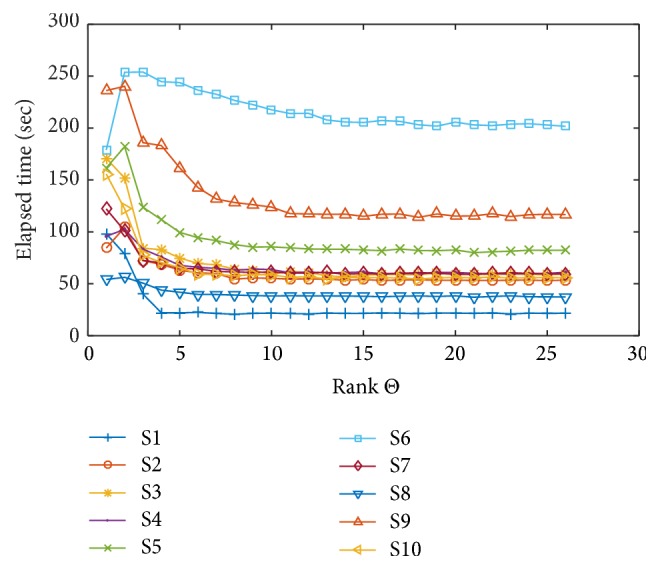
The change of elapsed time of convergence of optimization due to the change of low-rank constraint from 1 to 26 (full-rank) for all subjects.

The elapsed time of convergence of the low-rank constrained optimization is shown in Figure 4. The time gradually decreases reciprocally as the rank increases and reaches plateau at some rank constraint. The trend is very similar to that of mean classification error rate in Figure 3. It is also perceived that subject data with higher classification error rates tend to have longer convergence times.

**Figure 5:**
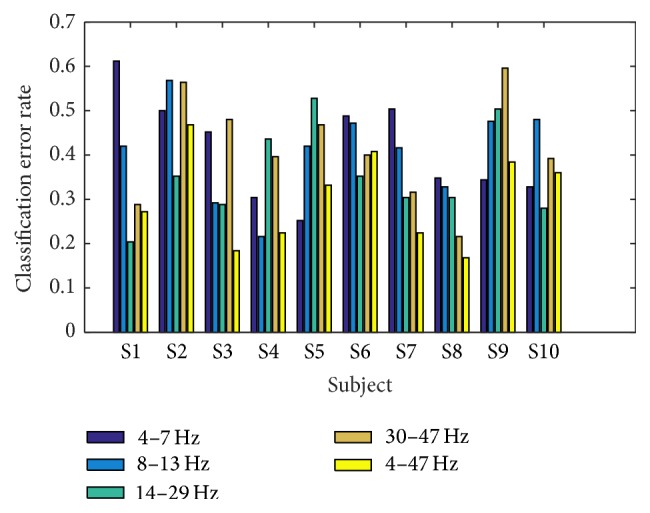
Classification error rate for different frequency bands of band-pass filter.

Figure 5 shows the change of mean classification error rate by changing the frequency band of band-pass filter to theta (4–7 Hz), alpha (8–13 Hz), beta (14–29 Hz), gamma (30–47 Hz), and wide band (4–47 Hz) in the preprocessing step. We use rank 6 for all the frequency bands. On average, better performance was obtained for beta and gamma bands compared with lower bands, that is, theta and alpha bands. The best performance was obtained when wide frequency band was used.

For comparative analysis with other methods, the spatial filters were calculated by CSP using identical preprocessed data. Since the proposed method used rank 6 constraint for analysis, six CSP filters were used for the alternative methods. CSP filters were selected automatically by three eigenvectors with the highest/lowest eigenvalues. We performed fivefold cross-validation with different classification algorithms, namely, ElasticNet, LDA, QDA, linear SVM (L-SVM), and SVM with RBF kernel (R-SVM). For all methods, we used identical feature vectors by employing selected CSP filters. Note that, for each round, the spatial filters were recalculated using only the training data.

**Figure 6:**
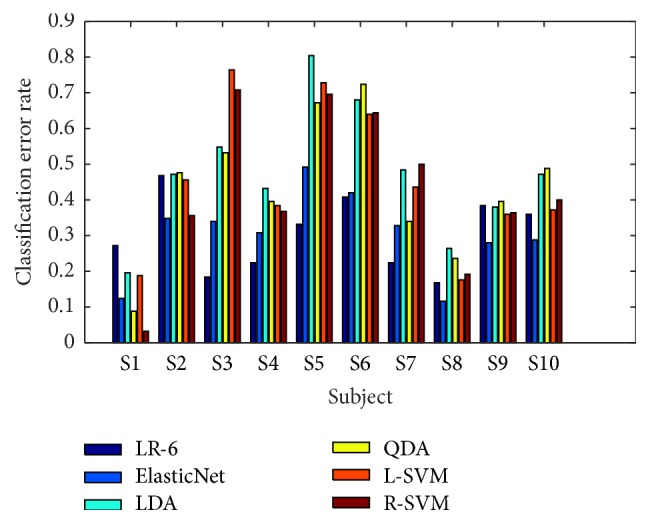
Comparative analysis of classification error rate to fix different methods; the proposed method with low-rank 6 (LR-6), elastic net (ElasticNet), linear discriminant analysis (LDA), quadratic discriminant analysis (QDA), linear SVM (L-SVM), and SVM with RBF kernel (R-SVM).

Figure 6 describes subjectwise comparison of the mean classification error rates of the proposed method with rank 6 constraint (LR-6) and the six conventional methods with CSP. Except subject “S1,” the proposed method achieved better or comparative results compared with the other methods.

Table 1 describes the comparison results. The classification error rates were obtained by averaging over subjects. The proposed method outperforms CSP-based LDA, QDA, L-SVM, and R-SVM methods and shows comparative performance against ElasticNet, the state-of-the-art method.

### 5.1. Discussion

If all the 23 subjects' data are used for analysis, the mean classification error rate was dropped from 0.302 (±0.103) to 0.412 (±0.131) when using the proposed method (LR-6). This is because the results of excluded subjects show below or just above chance level. The degradation of these results was common irrespective of methods including conventional methods. Therefore, these subjects data were deemed untrustworthy, so we manually select ten subjects for the analysis. The training/test data are non-i.i.d. because of the sliding window approach; that is, there are temporal correlations among neighboring data. But our assumption is that even if i.i.d. assumptions are violated, the proposed method would work well in practice.

The low-rank constrained linear model in (7) can be transformed as follows:

(15) f=b+Θ,Σ

(16) =b+∑i=1cσiTr⁡viuiTXXT

(17) =b+∑i=1cσiuiTXXTvi.

The last equation indicates that the spatial filters, {*u*_*i*_}_*i*=1_^*c*^ and {*v*_*i*_}_*i*=1_^*c*^, are applied to the covariant matrix of *X* from left and right, and the inner product of the spatially filtered signals is used to form the feature vector. The weighting coefficients of the feature vector correspond to singular values {*σ*_*i*_}_*i*=1_^*c*^. Note that spatial filters *u*_*i*_ and *v*_*i*_ are almost identical, possibly with different signs, due to the nature of the original linear model denoted by (7). Hence, it corresponds to computing the power of spatially filtered signals, similarly to CSP-based methods.

**Figure 7:**
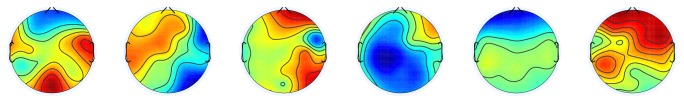
(Positive versus negative) topographies of six significant spatial filters obtained by *k*-mean clustering of all spatial filters estimated for all subjects by using rank 6 constraint. The color is mapped from −0.5 (blue) to +0.5 (red).

The topographies in Figure 7 represent the scalp maps of six representative spatial filters, which are obtained by *k*-mean clustering of estimated spatial filters for all subjects as shown in Figure 12. The spatial filters are defined by the left singular vectors of Θ. The color of topography is mapped from +0.5 (red) to −0.5 (blue).

**Figure 8:**
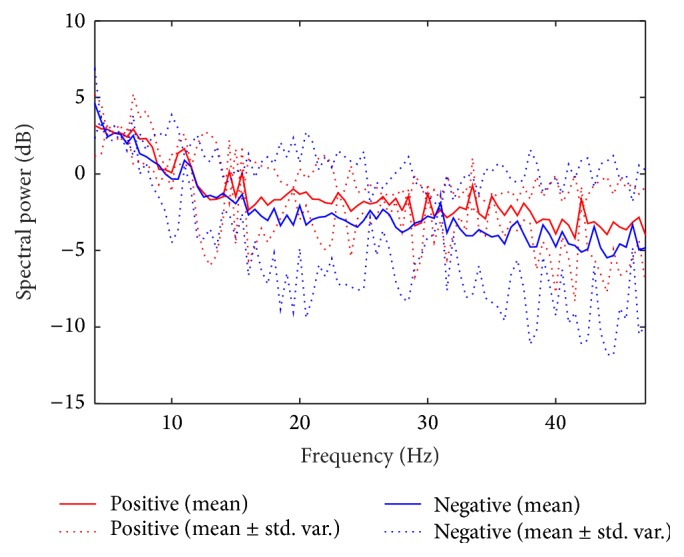
Comparison of mean PSDs between positive and negative. The mean PSD is calculated over all spatially filtered channels and subjects.

Figure 8 shows the difference of mean spectral power density (PSD) between positive and negative over all subjects. The mean PSD is calculated by averaging PSDs of all spatially filtered signals by using six spatial filters estimated over all subjects. Hence the mean PSD represents total average PSD of spatially filtered EEG signals. The dotted plots show the deviation from the mean. From this figure, we can observe that positive tends to have larger power than negative especially over beta and gamma frequency bands.

### 5.2. Valence versus Neutral

In order to further examine the differences between positive/negative valence and neutral, two-way binary classifications were conducted for positive versus neutral and negative versus neutral. For the analysis, we employed the proposed method with rank 6 constraint as described above for positive versus negative analysis. The preprocessing is exactly the same as before except for training/test data which is relevant to the target two classes. The data were band-pass filtered at 4–47 Hz.

**Figure 9:**
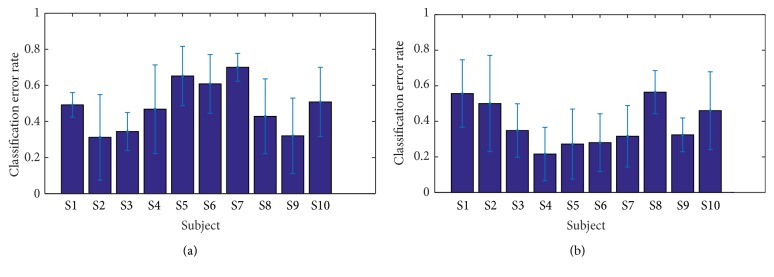
The figures show binary classification error rate of ten subjects in two cases: (a) positive versus neutral and (b) negative versus neutral. Mean classification error rates are (a) 0.483 ± 0.131 and (b) 0.384 ± 0.119.

Figure 9 describes the classification error rates of the two cases: positive versus neutral and negative versus neutral for the same subjects as before. The mean and std. variation of classification errors were as follows: positive versus neutral (0.483 ± 0.131) and negative versus neutral (0.384 ± 0.119). From this result, we notice that subjects with high classification performance for positive versus neutral case tend to have low performance for negative versus neutral case.

**Figure 10:**
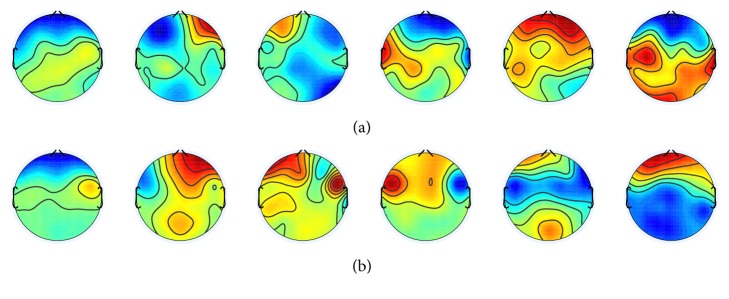
Topographies of six significant spatial filters obtained by *k*-mean clustering of all spatial filters estimated for all subjects by using rank 6 constraint. The color is mapped from −0.5 (blue) to +0.5 (red). (a) Positive versus neutral and (b) negative versus neutral.

Figure 10 shows the scalp maps of six representative spatial filters for (a) positive versus neutral and (b) negative versus neutral, which are obtained by *k*-mean clustering of estimated spatial filters as described for Figure 7. Figures 13 and 14 show the estimated spatial filters for all subjects for positive versus neutral and negative versus neutral, respectively.

As we described in Section 2, many researches suggest that hemispherical asymmetry over the frontal cortex is implicated for emotions and motivations. If the assumption is true, our hypothesis is that the scalp maps of estimated spatial filters for valence versus neutral will likely show asymmetrical patterns over the frontal lobe, as such spatial filters should increase inference accuracy.

Among the topographies in Figure 10, about half of them do show asymmetrical patterns over the frontal and left/right temporal lobe area. It is difficult but slightly observable that left or right lateralization corresponds to positive or negative valence as indicated by previous works [6, 7].

**Figure 11:**
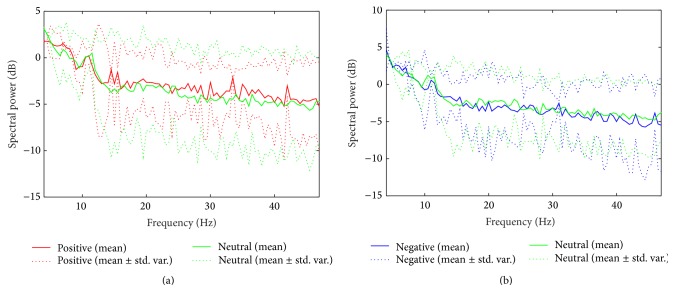
Comparison of mean PSDs between valence and neutral. The mean PSD is calculated over all spatially filtered channels and subjects. (a) Positive versus neutral and (b) negative versus neutral.

**Figure 12:**
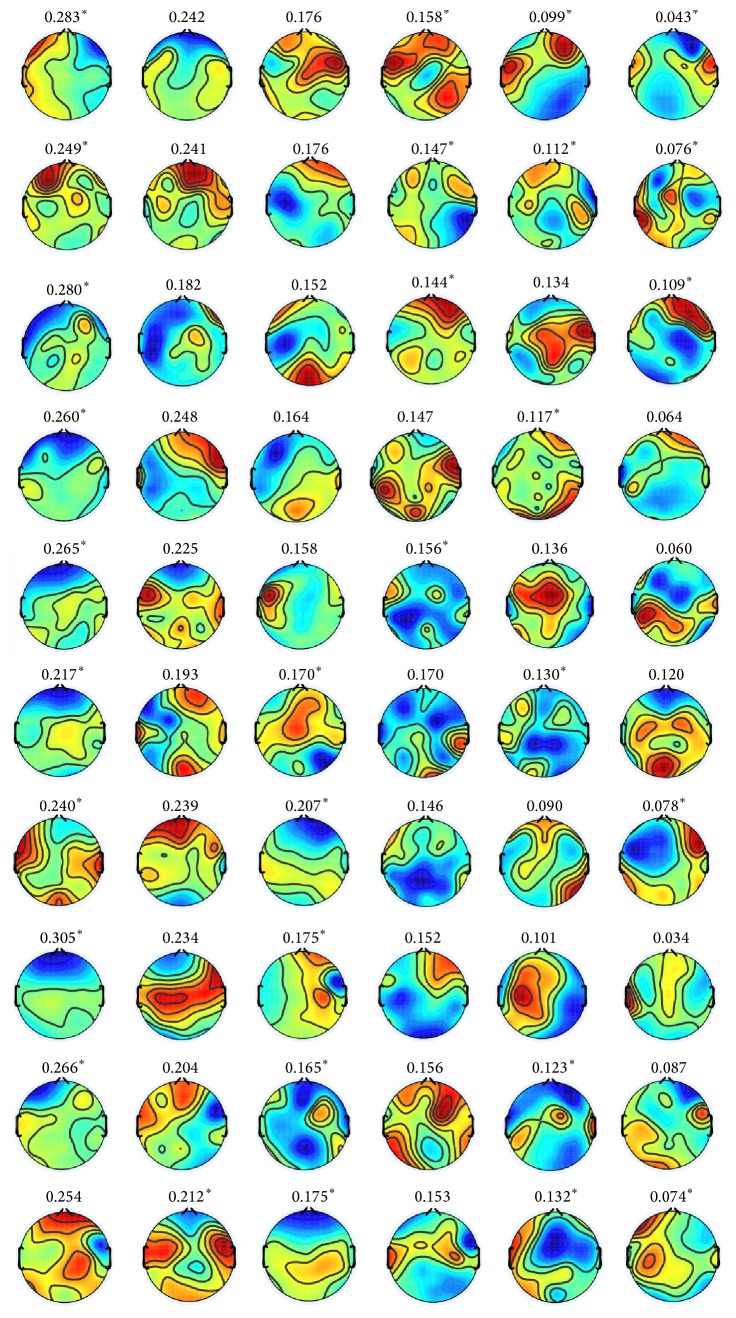
(Positive versus negative) scalp maps of spatial filters obtained by the proposed method with rank 6 constraint for all subjects. The row specifies subjects “S1”–“S10” from top to bottom. The column specifies six spatial filters defined by left singular vectors of Θ corresponding to the highest six eigenvalues from left to right. The number above each topography is the percentile of corresponding singular value. The superscript (*∗*) indicates that the pair of left and right singular vectors differs in sign.

**Figure 13:**
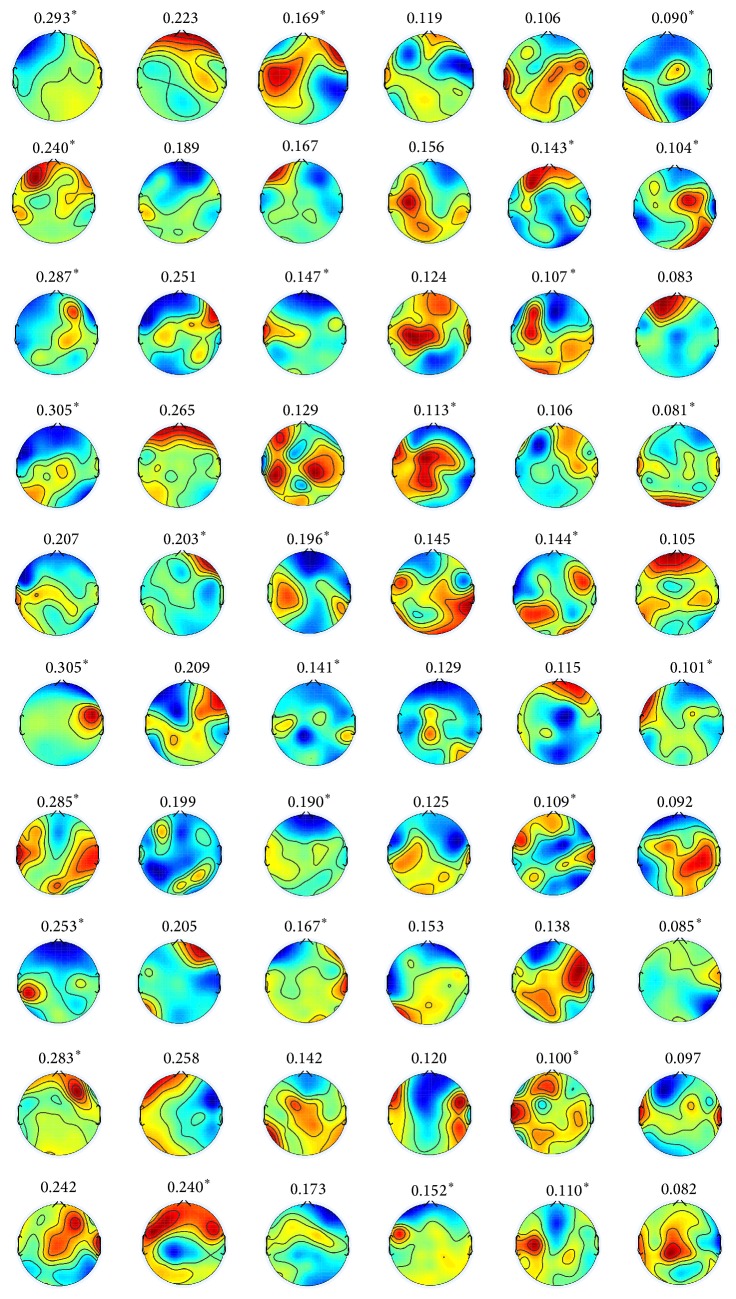
(Positive versus neutral) scalp maps of spatial filters obtained by the proposed method with rank 6 constraint for all subjects. The row specifies subjects “S1”–“S10” from top to bottom. The column specifies six spatial filters defined by left singular vectors of Θ corresponding to the highest six eigenvalues from left to right. The number above each topography is the percentile of corresponding singular value. The superscript (*∗*) indicates that the pair of left and right singular vectors differs in sign.

**Figure 14:**
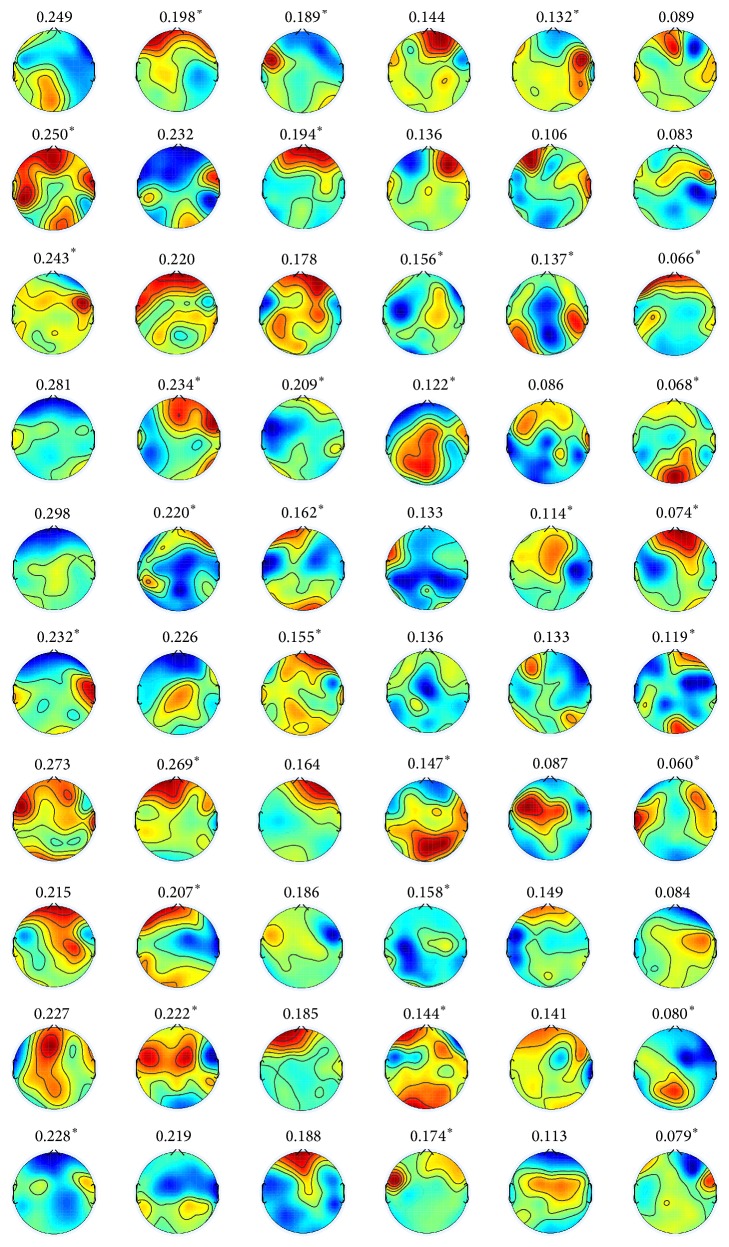
(Negative versus neutral) scalp maps of spatial filters obtained by the proposed method with rank 6 constraint for all subjects. The row specifies subjects “S1”–“S10” from top to bottom. The column specifies six spatial filters defined by left singular vectors of Θ corresponding to the highest six eigenvalues from left to right. The number above each topography is the percentile of corresponding singular value. The superscript (*∗*) indicates that the pair of left and right singular vectors differs in sign.

Figures 11(a) and 11(b) show the difference of mean PSD between positive/negative and neutral over all spatially filtered channels and subjects. The mean PSD is obtained similarly as positive versus negative case as shown in Figure 8.

From these figures, we can observe that positive has larger power than neutral in beta and gamma bands. On the other hand, negative has similar or slightly lower power than neutral in those bands.

## 6. Conclusion

In this paper, a fixed low-rank spatial filter estimation for BCI systems was proposed with an application of emotion recognition induced by movies. The proposed approach unifies such tasks as feature extraction, feature selection, and classification, which are often independently tackled in a “bottom-up” manner, under a regularized loss minimization problem. We explicitly derived the loss function from the conventional BCI approach and solved its minimization by optimization with a nonconvex fixed low-rank constraint.

The proposed method derived from “top-down” approach incorporates spatial filter estimation in the predictive model. Hence by focusing on the prediction performance with suitably chosen regularizer, such as fixed low-rank in our model, it induces sparse decomposition of the signal which corresponds to conventional feature extraction. Hence, it implicitly estimates optimal spatial filters of the best inference model under the assumption. The result of comparative analysis shows that the proposed method is competitive and has equivalent performance to the best CSP-based alternative.

In the discussion, we show that about half of the significant scalp maps of spatial filters estimated for positive versus negative do show asymmetrical patterns over the frontal and temporal lobe, which agree with the previous research works; that is, asymmetrical patterns over frontal cortex are implicated for emotions and motivations. We also observe that positive state tends to exhibit larger power than negative state over beta and gamma frequency bands. The opposite lateralization of hemispherical activity is weakly admitted for positive and negative cases.

There are some directions for future work and some suggestions for improving performance. First, extending the proposed method to multiclass classification is required to recognize variety of emotional states. Second, source space analysis might be useful to further investigate subcortical activities of emotions. Lastly, obtaining genuine training/test data is of primal importance especially for BCIs depending on interoceptive inputs like thoughts and emotions. One possible solution is to evaluate labels based on ratings of participants.

## Figures and Tables

**Table 1:** Comparative results of classification error rate. LR-6 specifies the proposed method with rank 6 constraint. In the leftmost column, avg. and std. var. specify mean and standard deviation of classification error rate over folds and subjects.

	LR-6	ElasticNet	LDA	QDA	L-SVM	R-SVM
Avg.	0.302	0.304	0.473	0.435	0.450	0.426
Std. var.	0.103	0.116	0.179	0.190	0.204	0.218

## References

[1] Blankertz B., Tomioka R., Lemm S., Kawanabe M., Müller K.-R. (2008). Optimizing spatial filters for robust EEG single-trial analysis. *IEEE Signal Processing Magazine*.

[2] Tomioka R., Müller K.-R. (2010). A regularized discriminative framework for EEG analysis with application to brain-computer interface. *NeuroImage*.

[3] Wang X.-W., Nie D., Lu B.-L. (2014). Emotional state classification from EEG data using machine learning approach. *Neurocomputing*.

[4] Daly I., Malik A., Hwang F. (2014). Neural correlates of emotional responses to music: an EEG study. *Neuroscience Letters*.

[5] Kandel E. R., Schwartz J. H., Jessell T. M. (2000). *Principles of Neural Science*.

[6] Harmon-Jones E. (2003). Clarifying the emotive functions of asymmetrical frontal cortical activity. *Psychophysiology*.

[7] Davidson R. J., Fox N. A. (1982). Asymmetrical brain activity discriminates between positive and negative affective stimuli in human infants. *Science*.

[8] Lotte F., Guan C. (2011). Regularizing common spatial patterns to improve BCI designs: unified theory and new algorithms. *IEEE Transactions on Biomedical Engineering*.

[9] Boyd S., Parikh N., Chu E., Peleato B., Eckstein J. (2011). Distributed optimization and statistical learning via the alternating direction method of multipliers. *Foundations and Trends in Machine Learning*.

[10] Samson A. C., Kreibig S. D., Soderstrom B., Wade A. A., Gross J. J. (2015). Eliciting positive, negative and mixed emotional states: a film library for affective scientists. *Cognition and Emotion*.

[11] Gómez-Herrero G., De Clercq W., Anwar H. Automatic removal of ocular artifacts in the EEG without an EOG reference channel.

